# Cloning of the Cryptochrome-Encoding *PeCRY1* Gene from *Populus euphratica* and Functional Analysis in *Arabidopsis*

**DOI:** 10.1371/journal.pone.0115201

**Published:** 2014-12-12

**Authors:** Ke Mao, Libo Jiang, Wenhao Bo, Fang Xu, Rongling Wu

**Affiliations:** Center for Computational Biology, College of Biological Science and Technology, Beijing Forestry University, Beijing 100083, China; Ruhr-University Bochum, Germany

## Abstract

Cryptochromes are photolyase-like blue/UV-A light receptors that evolved from photolyases. In plants, cryptochromes regulate various aspects of plant growth and development. Despite of their involvement in the control of important plant traits, however, most studies on cryptochromes have focused on lower plants and herbaceous crops, and no data on cryptochrome function are available for forest trees. In this study, we isolated a cryptochrome gene, *PeCRY1*, from Euphrates poplar (*Populus euphratica*), and analyzed its structure and function in detail. The deduced PeCRY1 amino acid sequence contained a conserved N-terminal photolyase-homologous region (PHR) domain as well as a C-terminal DQXVP-acidic-STAES (DAS) domain. Secondary and tertiary structure analysis showed that PeCRY1 shares high similarity with AtCRY1 from *Arabidopsis thaliana*. *PeCRY1* expression was upregulated at the mRNA level by light. Using heterologous expression in *Arabidopsis*, we showed that PeCRY1 overexpression rescued the *cry1* mutant phenotype. In addition, PeCRY1 overexpression inhibited hypocotyl elongation, promoted root growth, and enhanced anthocyanin accumulation in wild-type background seedlings grown under blue light. Furthermore, we examined the interaction between PeCRY1 and AtCOP1 using a bimolecular fluorescence complementation (BiFc) assay. Our data provide evidence for the involvement of PeCRY1 in the control of photomorphogenesis in poplar.

## Introduction

Light is one of the most important environmental factors for plants as it provides the source of energy to sustain plant life [1]. Light is also a key signal controlling virtually every aspect of plant growth and development [2]. As a consequence, plants have the ability to sense multiple parameters of ambient light signals including light quantity (fluence), quality (wavelength), direction, and duration [1]. Light promotes the developmental transition from skotomorphogenesis to photomorphogenesis in plants through the combinatorial interaction of diverse sensory photoreceptors, which are classified based on the wavelength of light they perceive [3]. Light signals are perceived through at least four distinct families of photoreceptors including red/far-red (600–750 nm) light receptor phytochromes [4], blue/UV-A (320–500 nm) light receptor phototropins [5], cryptochromes [6], [7], ZEITLUPE (ZTL), FLAVIN BINDING, KELCH REPEAT, F-BOX1 (FKF1), and LOV KELCH PROTEIN2 (LKP2) and the UV-B (280–320 nm) light receptor UVR8 [8].

Cryptochromes are photolyase-like blue/UV-A light receptors that evolved from photolyases, a class of blue light-activated microbial DNA repair enzymes [9], [10], but rather than repairing DNA, cryptochromes regulate numerous aspects of growth and development in a wide range of organisms from bacteria to humans [11], [12]. In plants, cryptochromes mediate blue light-dependent inhibition of hypocotyl elongation, deetiolation responses, control of vegetative growth, flowering initiation, anthocyanin accumulation, regulation of gene expression, and the maintenance of plant endogenous rhythms [7].

Cryptochrome was first identified in *Arabidopsis thaliana* during the molecular characterization of the *Arabidopsis* mutant *hy4*, which exhibited elongated hypocotyls when grown in blue light [13]. The *hy4* mutant was subsequently renamed *cry1*, defining the *CRY1* gene encoding cryptochrome 1 [14], which mediates deetiolation in response to blue light. Subsequently, a second member of the *Arabidopsis* cryptochrome family, CRY2, which primarily regulates photoperiodic flowering [15], was identified by screening *Arabidopsis* cDNA libraries with *CRY1* cDNA probes [16], [17]. A third *CRY* family member, CRY-DASH, from *Arabidopsis* has been characterized [18] and exhibits single-stranded DNA-specific photolyase activity [19]. Since the discovery of the first cryptochrome in *Arabidopsis*, this type of photoreceptor has been found widely in organisms ranging from bacteria to humans [11], [12]. In addition to *Arabidopsis*, cryptochromes have been studied in various photosynthetic species including algae [20], moss [21], fern [22], tomato [23], [24], rapeseed [25], pea [26], rice [27], [28], and apple [29], [30].

All cryptochromes are composed of two major domains, the N-terminal photolyase-homologous region (PHR) domain of approximately 500 residues and the CRY C-terminal extension (CCE) domain of various lengths. The PHR domain is required to bind the two chromophores flavin adenine dinucleotide (FAD) [31]–[33] and 5,10-methenyltetrahydrofolate (MTHF) [19], [34], whereas CCE is a cryptochrome effector domain [35] that governs the signaling activity of photoactivated cryptochromes.

The CRY1-PHR of *Arabidopsis* contains two subdomains similar to photolyase and CRY-DASH: an N-terminal α/β subdomain (residues 13–139) connected via a loop to the C-terminal α-helical subdomain (residues 217–495) [36]. The α/β subdomain has a five-stranded, parallel β-sheet flanked by four α-helices and a 3_10_ helix (a less common α-helix with 3.0 residues per turn instead of 3.6 residues per turn) resembling a dinucleotide-binding domain. The CCE domains of plant cryptochromes are intrinsically unstructured [9] with little sequence similarity, and the cryptochromes of different plant species are distinguished mainly by their CCE domains [35]. However, plant cryptochromes from different species share a common DQXVP–acidic- STAESSS (DAS) sequence motif in their CCE domains.

Previous studies have indicated that *Arabidopsis CRY1* and *CRY2* were expressed ubiquitously in all cells and organ types examined [14], [15], [37]. The cryptochrome genes of some plant species exhibit expression patterns in different tissues similar to those in *Arabidopsis* and are expressed at higher levels during the early stages of development. As blue light receptors, plant *CRY* mRNA levels are regulated dramatically by light. For example, the expression of both *Arabidopsis CRY1* and *CRY2* is regulated by the circadian clock with peaks during the light phase and troughs during the dark phase [37].

In addition to *CRY* gene regulation at the transcript level, CRY protein levels are also regulated by light. *Arabidopsis* CRY2 protein is light-labile, but CRY1 protein is light-stable. Most plant CRY proteins are nuclear-localized; both *Arabidopsis* CRY1 and CRY2 accumulate in the nucleus, but AtCRY1 can also be detected in the cytosol of seedlings grown under either dark or light conditions [38]. Nuclear CRY1 protein has been shown to be responsible for blue light inhibition of hypocotyl elongation, whereas cytosolically localized CRY1 mediates blue light stimulation of cotyledon expansion and root elongation [38]. In contrast, CRY2 appears to complete its posttranslational life cycle in the nucleus [39].

Although the signal transduction mechanism of plant cryptochromes is not fully understood, cryptochromes are generally thought to interact with signaling proteins to regulate gene expression [1], [11], [12]. Results of DNA microarray analyses suggested that the expression of approximately 5–25% of the genes in the *Arabidopsis* genome changes in response to blue light with most of the changes mediated by CRY1 and CRY2 [40]–[43]. In *Arabidopsis*, both CRY1 and CRY2 were shown to interact directly with the constitutive photomorphogenic 1 (COP1) protein and inhibit its E3 ubiquitin ligase activity to prevent the degradation of COP1 substrates such as the long hypocotyl in far-red 1 (HFR1) [44] and CONSTANS (CO) proteins [45]. Therefore, the CRY proteins stabilize transcription factors that can regulate gene expression and promote photomorphogenesis in *Arabidopsis*.

Despite their involvement in the control of important plant traits such as plant height, root length, and flowering time, most studies on cryptochromes have focused only on lower plant species and herbaceous crops [30], [46]. The cryptochromes of forest tree species have not been extensively characterized. In this study, we isolated the *PeCRY1* gene encoding a blue light receptor from the desert species *Populus euphratica*, analyzed its structure and relationship with *CRY* genes from other species, and substantiated the role of *PeCRY1* in regulating plant height, root length, and anthocyanin accumulation. Our results indicate that *PeCRY1* plays an important role in the regulation of growth and development of Euphrates poplar.

## Results

### Cloning of a Full-Length *PeCRY1* cDNA

To isolate a full-length cDNA sequence of the Euphrates poplar cryptochrome gene *PeCRY1*, expressed sequence tag (EST) clones with similarity to *Arabidopsis* cryptochrome 1 were identified by analysis of Euphrates poplar dbEST sequences in the National Center for Biotechnology Information (NCBI) database. A 581-bp *PeCRY1* fragment was isolated from Euphrates poplar leaves and 5′/3′-RACE extension methods were used to obtain the missing *PeCRY1* sequences. The EST sequence (AJ768957) and two fragments were then combined based on analysis using DNAman software (Lynnon Corp., Pointe-Claire, QB, Canada) to obtain a full-length *PeCRY1* sequence. The full-length *PeCRY1* cDNA sequence contained a 2,046-bp open reading frame (ORF). The *PeCRY1* ORF encoded a protein of 681 amino acids with a calculated mass of 76.9 kDa as predicted using DNAstar software. The deduced protein was basic with an isoelectric point (pI) of 5.64 as predicted using the DNAman software.

### PeCRY1 Amino Acid Sequence Analysis

**Figure 1:**
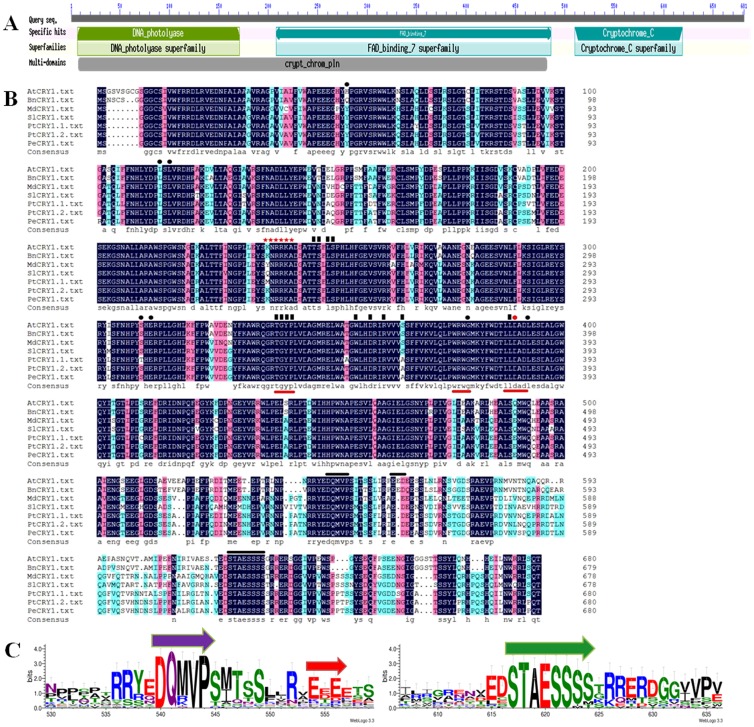
PeCRY1 protein sequence analysis. (A) Structural domains of the PeCRY1 protein. Analysis of protein sequences in the National Center for Biotechnology Information (NCBI) database was performed using the CD-search software. (B) Amino acid sequence alignment of cryptochrome proteins from *Arabidopsis*, winter rape, apple, tomato, European aspen, and Euphrates poplar. The alignment was constructed using DNAman version 5.2.2 software. Identical residues are highlighted by black boxes. Red lines under the sequences indicate the TGYP, WRWK, and LLDAD motifs. Black lines above the sequences indicate the DQMVP-E/D-STAESS (DAS) domain located in the C-terminal region. Residues that interact with FAD and MTHF are indicated by black rectangles and black dots, respectively. A nuclear localization signal (NLS) predicted using NLStradamus software is indicated by red stars. (C) DAS domain sequence logos. The sequence alignment of the domains was generated using ClustalX, and conserved motif logos were created using the WebLogo program (http://weblogo.threeplusone.com/).

Sequence analysis using the CD-search program to explore the NCBI database (http://structure.ncbi.nlm.nih.gov/Structure/cdd/wrpsb.cgi) revealed that the PeCRY1 amino acid sequence contained a well-conserved N-terminal PHR domain, which is required for binding of the chromophore flavin adenine dinucleotide (FAD) [25] and a C-terminal CCE region containing a DAS domain [35] (Fig. 1A).

AtCRY1 associates with two cofactors, the catalytic cofactor (FAD) and a light-harvesting cofactor (MTHF), in the same manner as Type I photolyases [31], [47]. Twelve of 13 amino acids in AtCRY1 predicted to interact with FAD were conserved in PeCRY1 with the exception of the serine at position 359, which was replaced by an alanine (Fig. 1B). In addition, six of seven identical amino acid residues (His-52 was replaced by Gln) known to interact with MTHF were also conserved in PeCRY1 (Fig. 1B). In addition, the TGYP motif, which is conserved in all Type I photolyases and forms a part of the FAD-binding domain [47]; the WRWG motif, which is well conserved among photolyases and cryptochromes [14], [48]; and the LLDAD motif, which is a conserved region of the FAD-binding pocket of cryptochromes [31], [49], were also present in PeCRY1 (Fig. 1B).

Three important motifs, known collectively as the DAS domain (Fig. 1B), including the DQXVP motif of unknown function, an acidic region (represented by E and D), and the STAESS motif implicated in the interaction with phytochrome A (phyA) (Fig. 1B, C) [50], were present in the PeCRY1C-terminal region and conserved, although the overall similarity was low.

### Structural Analysis of the PeCRY1 Amino Acid Sequence

**Figure 2:**
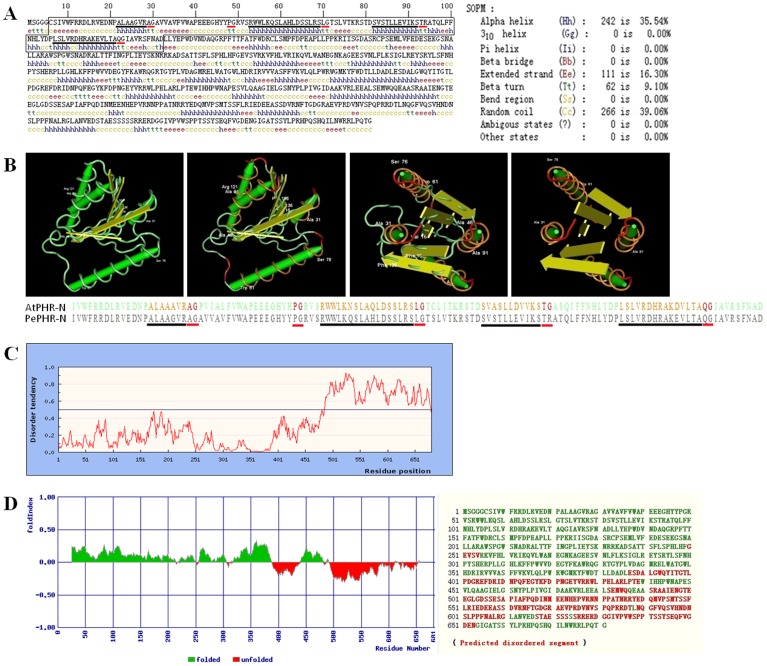
Structural analysis of the PeCRY1 protein. (A) The secondary structure of PeCRY1 solved by the self-optimized prediction method (SPOM). The black box indicates the PeCRY1 PHR-N domain corresponding to that of AtCRY1. Black and red lines beneath the sequence indicate the α-helices and the β-turns in the PHR-N domain. (B) Comparison of the predicted three-dimensional structures of PeCRY1 and AtCRY1 using Cn3D software. Orange and red segments in the images correspond to the sequences marked by black and red lines, respectively, in (A). (C) Local disorder tendency of the PeCRY1 CCE domain based on an estimated-amino-acid-pairwise-energy-content analysis using IUPred software. (D) The fold disordering character of PeCRY1 predicted using FoldIndex software.

The PeCRY1 secondary structure was solved using the self-optimized prediction method (SOPM, http://npsa-pbil.ibcp.fr/cgi-bin/npsa_automat.pl?page=npsa_sopm.html) [51]. PeCRY1 consists of α-helices (242 aa, 35.54%), β-turns (62 aa, 9.10%), extended strands (111 aa, 16.30%), and random coil (266 aa, 39.06%) regions (Fig. 2A). The α-helices and β-turns were distributed randomly throughout the PeCRY1 polypeptide (Fig. 2A).

The α/β subdomain of CRY1-PHR in *Arabidopsis* has five-stranded, parallel β-sheets flanked by four α-helices and a 3_10_ helix, which collectively resemble a dinucleotide-binding domain. Although a 3_10_ helix was not present in PeCRY1, four α-helices were identified in the same region as in AtCRY1 using the SOPM method (Fig. 2A). Moreover, we identified five β-turns in the α/β subdomain (Fig. 2A), similar to AtCRY1 using the Cn3D macromolecular structure viewer software (PDB: 1U3C_A), suggesting that these β-turns might form parallel β-sheets in this region and be flanked by the four α-helices as in AtCRY1 (Fig. 2B).

CRY proteins from different plant species are distinguished mainly by C-terminal extensions [29]. Previous studies suggested that the CCE domain is important for cryptochrome function in plants [14] and is intrinsically unstructured [12]. Analysis using circular dichroism and nuclear magnetic resonance (NMR) demonstrated that the CCE domains of *Arabidopsis* CRY1 and human CRY2 are unstructured [12]. We confirmed the intrinsically unstructured nature of the PeCRY1 CCE domain based on an analysis of estimated amino acid pairwise energy content using IUPred software (http://iupred.enzim.hu/) (Fig. 2C). In addition, we investigated the fold disordering character of PeCRY1 using FoldIndex software (http://bip.weizmann.ac.il/fldbin/findex) [52]. We identified seven disordered regions in the PeCRY1 sequence with the longest disordered region (97 aa) located in the CCE domain with a total of 218 disordered amino acid residues (Fig. 2D).

**Figure 3:**
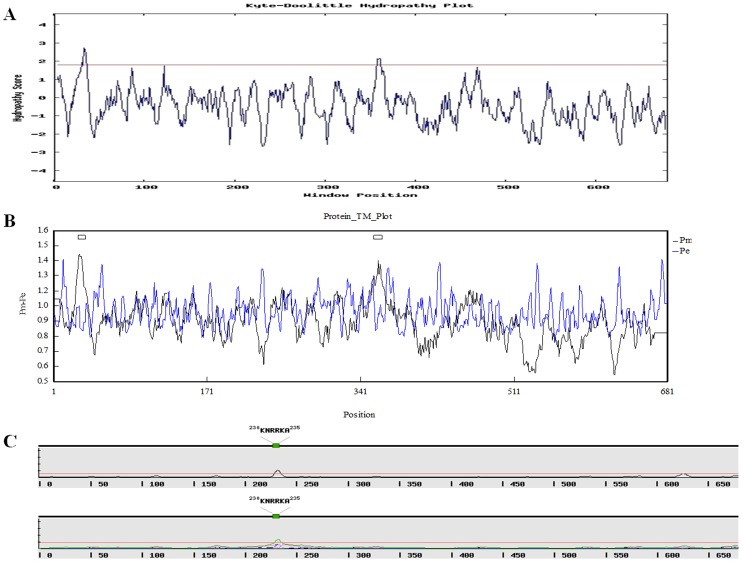
Hydrophilicity/hydrophobicity analysis and PeCRY1 transmembrane domain prediction. (A) Hydrophilicity/hydrophobicity analysis of PeCRY1 performed using the Kyte and Doolittle method. A score of 1.8 is indicated by the red line. (B) PeCRY1 transmembrane domain prediction using DNAman version 5.2.2 software with default options. Predicted transmembrane regions are indicated by boxes above the profile. (C) PeCRY1 nuclear localization signal (NLS) prediction using a simple hidden Markov model (HMM) and NLStradamus software. The analysis was performed using both a 2-state HMM dynamic model (above) and a 4-state HMM static model (below).

We also investigated the hydrophilicity/hydrophobicity of PeCRY1 using the Kyte and Doolittle method (http://gcat.davidson.edu/DGPB/kd/kyte-doolittle.htm) [53]. The majority of PeCRY1 amino acids were hydrophilic, and almost all of the C-terminal region amino acids were hydrophilic, indicating that PeCRY1 is a hydrophilic protein (Fig. 3A). In addition, two PeCRY1 transmembrane domains with scores above 1.8 were also predicted using the Kyte and Doolittle method (Fig. 3A). To confirm this result, we examined the PeCRY1 transmembrane domains using DNAman software, which identified two possible transmembrane domains in PeCRY1, the first located at amino acids 23–40 and the second at amino acids 351–369 (Fig. 3B), in agreement with the results obtained using the Kyte and Doolittle method.

Most plant CRY proteins are thought to be nuclear localized proteins. Both *Arabidopsis* CRY1 and CRY2 accumulate in the nucleus [35]. Using NLStradamus software (http://www.moseslab.csb.utoronto.ca/NLStradamus/), a simple hidden Markov model (HMM) for nuclear localization signal prediction, we identified a nuclear localization signal (NLS) sequence “KNRRKA” in PeCRY1 at amino acids 230–235 (Fig. 3C), indicating that PeCRY1 is a nuclear localized protein. Analysis with the NLStradamus software using both 2-state HMM dynamic and 4-state HMM static models gave the same results.

### Relationship of PeCRY1 to Other Cryptochromes

**Figure 4:**
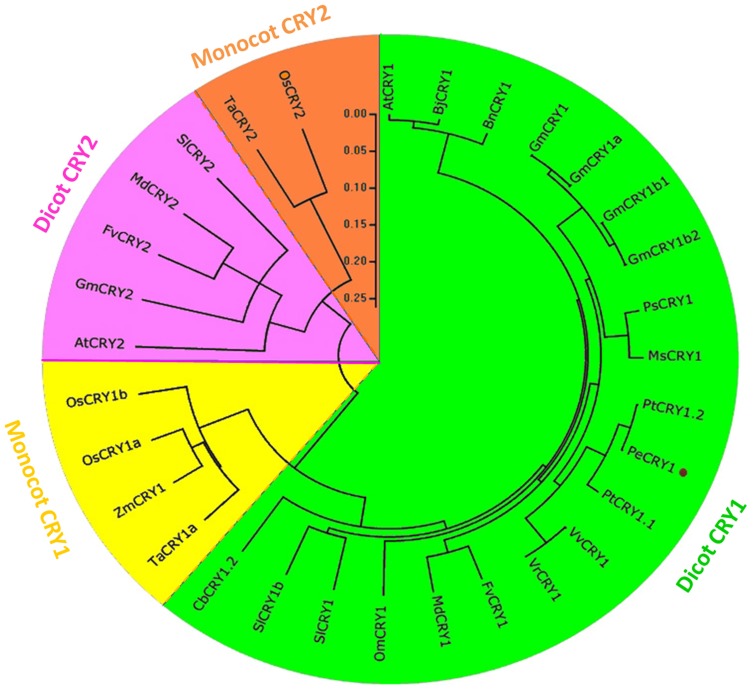
Phylogram of plant cryptochromes. CRY amino acid sequences from 14 diverse plant species were obtained from the NCBI database. The alignment was constructed using ClustalX and the phylogenetic tree was constructed using the neighbor-joining method of MEGA version 4.1 software. Each node corresponds to a number indicating the bootstrap value for 1000 replicates. The scale bar represents 0.05 substitutions per sequence position. PeCRY1 is denoted by a red dot. *At*, *Arabidopsis thaliana*; *Bj*, *Brassica juncea*; *Bn*, *Brassica napus*; *Gm*, *Glycine max*; *Ps*, *Pisum sativum*; *Ms*, *Medicago sativa*; *Pt*, *Populus tremula*; *Pe*, *Populus euphratica*; *Vv*, *Vitis vinifera*; *Vr*, *Vitis riparia*; *Fv*, *Fragaria vesca*; *Md*, *Malus domestica*; *Om*, *Orobanche minor*; *Sl*, *Solanum lycopersicum*; *Cb*, *Chrysanthemum boreale*; *Ta*, *Triticum aestivum*; *Zm*, *Zea mays*; *Os, Oryza sativa.*

To analyze the phylogenetic relationship between PeCRY1 and the CRY proteins from other plant species, we performed a phylogenetic analysis of 30 plant cryptochromes representing 14 diverse species using the Mega4.1 program and the Clustal method (Fig. 4). The phylogenetic tree indicated obvious boundaries between the dicotyledonous and monocotyledonous CRY1 proteins. PeCRY1 grouped in the dicot CRY1 clade and was most closely related to the PtCRY1.1 (JN235115.1) and PtCRY1.2 (JN235116.1) CRY proteins of European aspen and the VvCRY1 (EU188919.1) and VrCRY1 (ABX80391.1) CRY proteins of grape, all of which clustered in the same clade. In contrast, PeCRY1 was most distantly related to the CRY1 proteins of monocotyledonous species, including wheat TaCRY1a (ABX58028.1), maize ZmCRY1 (AFW71952.1), and rice OsCRY1a (BAB70686.1) and OsCRY1b (BAB70688.2). In addition, the phylogenetic tree indicated that the plant CRY1 and CRY2 proteins formed in different groups with clear boundaries between dicotyledonous and monocotyledonous species (Fig. 4), demonstrating the consistency of plant evolution.

### *PeCRY1* Expression Analysis

**Figure 5:**
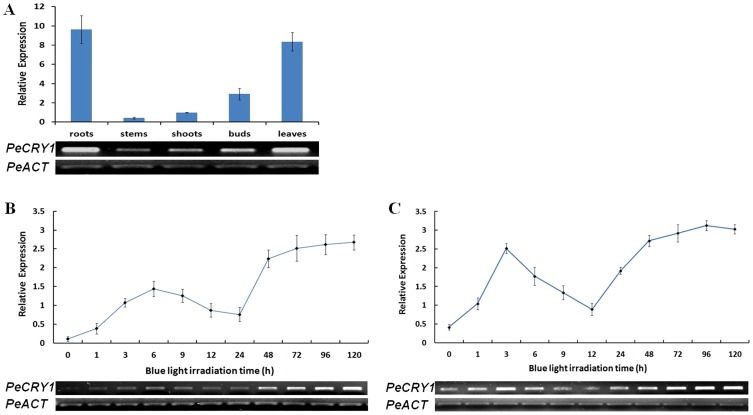
Analysis of *PeCRY1* expression using semiquantitative and real-time quantitative RT-PCR. (A) *PeCRY1* transcript levels in different tissues. (B) and (C) *PeCRY1* transcript levels in cultured seedlings or callus with different durations of blue light irradiation. Poplar actin (*PeACT*) expression was used as an internal control.

Previous studies showed that cryptochrome genes such as *AtCRY1* were expressed ubiquitously in all cell types and organs examined [14], [15], [37] and that they regulated different aspects of plant growth and development [7]. To examine whether *PeCRY1* transcript levels were tissue-specific, we performed semiquantitative and real-time quantitative reverse transcription (RT)-PCR analysis using total RNAs obtained from different tissues including roots, stems, shoots, buds, and leaves. *PeCRY1* was expressed in all of the tissues we examined and the expression levels varied among the tissues (Fig. 5A). *PeCRY1* expression was highest in the roots and leaves, followed by the buds and shoots with only weak expression detected in the stems.

As blue light receptors, the expression of cryptochrome genes such as *AtCRY1* and *AtCRY2* is regulated by blue light [35]. To study the effect of blue light on *PeCRY1* expression, cultured Euphrates poplar seedlings were grown in darkness for 3 days and then exposed to blue light for 0–120 h. Upon irradiation of the seedlings with blue light, *PeCRY1* exhibited low basal expression followed by a clear increase in transcript levels with extended irradiation time (Fig. 5B). The *PeCRY1* transcript levels reached a peak at 6 h, then decreased to a minimum at 24 h. Subsequently, transcript levels increased markedly to a peak at 72 h and remained stable through 120 h. Although the seedlings were treated with continuous blue light, the *PeCRY1* expression pattern exhibited a circadian rhythm during the first 24 h (Fig. 5B), similar to the observation in previous studies that *CRY* transcript levels exhibit an oscillation period of almost 24 h [26], [37]. To confirm the expression pattern of *PeCRY1* under blue light, we also analyzed *PeCRY1* expression using Euphrates poplar callus under the same light conditions with results similar to those obtained from the seedlings. However, the relative *PeCRY1* background expression was higher and responded more rapidly to blue light in the callus cells than in the cultured seedlings with a expression peak at 3 h, and the transcript rhythm cycle was within 12 h (Fig. 5C).

### Functional Complementation of the *Arabidopsis cry1* Mutant by *PeCRY1*

**Figure 6:**
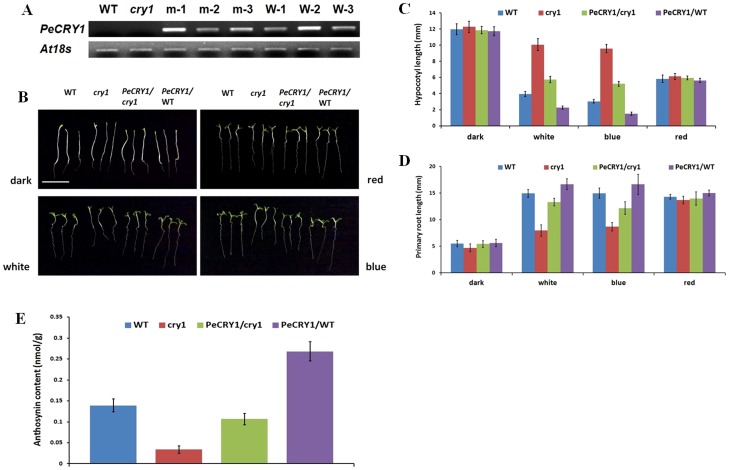
Phenotypes of wild-type (WT), *cry1*-mutant, *PeCRY1*-transgenic *cry1*-mutant, and *PeCRY1*-transgenic WT plants. (A) *PeCRY1* transcript levels in WT, *cry1* mutant, and transgenic lines. m-1, m-2, and m-3, three *cry1* mutant lines transformed with *PeCRY1*; W-1, W-2, and W-3, three WT lines transformed with *PeCRY1*. (B) Phenotypes of WT, *cry1*-mutant, and the two transgenic lines (m-1 and W-2) grown under dark, white, blue, and red light. Scale bar represents 1 cm. (C) and (D) Hypocotyl and primary root lengths of WT, *cry1*-mutant, and the two transgenic lines grown under dark, white, blue, and red light. (E) Anthocyanin content of WT, *cry*1-mutant, and the two transgenic lines grown under blue light.

To investigate the function of *PeCRY1* in plants, we conducted a functional complementation assay using an *Arabidopsis cry1* mutant. *PeCRY1* was introduced into a pRI vector (pRI 101-AN) with expression driven by a CaMV-35S promoter followed by a 58-bp *AtADH* 5′UTR enhancer (35S::PeCRY1) and transformed into wild-type (WT; Columbia ecotype) and *cry1*-mutant *Arabidopsis* backgrounds using the floral dip method [54]. After repeated selection on kanamycin and PCR screening for the presence of the transgene, at least three transformants of each background were obtained. Transgene expression levels were determined by semiquantitative RT-PCR to verify successful transformation (Fig. 6A). Two transgenic lines, m-1 and W-2, with high levels of PeCRY1 expression were chosen to compare the phenotypes of the WT, *cry1* mutant, *cry1* mutant transformed with *PeCRY1*, and *PeCRY1* overexpressing lines.

### *PeCRY1* Inhibits Hypocotyl Elongation in *Arabidopsis*

Previous research indicated that *AtCRY1* plays a prominent role in the inhibition of hypocotyl elongation [14] and that many other plant cryptochrome genes also inhibit hypocotyl elongation [23], [24], [29], [30]. To determine if *PeCRY1* inhibits hypocotyl elongation, we measured the hypocotyl lengths of the four types of *Arabidopsis* lines. When the seedlings were grown in complete darkness, the hypocotyl lengths did not differ significantly among the four genotypes (Fig. 6B, C). However, when grown under white light, the *cry1* mutant lines exhibited reduced inhibition of hypocotyl elongation, which was restored by transformation of the *cry1* mutant with *PeCRY1*. The *PeCRY1*-overexpressing lines exhibited the shortest hypocotyl lengths of the three genotypes (Fig. 6B, C). To examine whether the inhibitory effect was specific to blue light, seedlings of the four genotypes were grown under continuous blue or red light. Under blue light, the hypocotyls of *cry1* mutant and WT plants transformed with *PeCRY1* were shorter than those of the untransformed *cry1* mutant or WT plants. The hypocotyl lengths were not significantly different among the four *Arabidopsis* types when grown under red light, indicating that the inhibitory effects of *PeCRY1* were much less dramatic under red light (Fig. 6B, C). These results demonstrated that *PeCRY1* exhibited the same function as *AtCRY1* in the inhibition of hypocotyl elongation in plants.

### *PeCRY1* Promotes Root Elongation in *Arabidopsis*

Similar to the differences in hypocotyl elongation, primary root lengths were clearly different among the four genotypes when grown under light with the root lengths of the *cry1* mutants being significantly shorter than those of WT and *PeCRY1* transgenic plants (Fig. 6B, D). *AtCRY1* has been shown to promote root elongation under blue light conditions, while *cry1* mutant seedlings exhibit decreased root elongation [2]. In this study, we showed that *PeCRY1* also promoted root elongation. Transformation with *PeCRY1* complemented the reduced root elongation phenotype of the *cry1* mutant and WT plants transformed with *PeCRY1* had the longest roots. These results demonstrated that *PeCRY1* can regulate plant root elongation and that this regulation is more sensitive to blue light, similar to *AtCRY1*.

### PeCRY1 Increases Anthocyanin Accumulation in *Arabidopsis*

Previous studies showed that anthocyanin accumulation decreased markedly in the *Arabidopsis cry1* mutant and increased significantly in *Arabidopsis* seedlings overexpressing *AtCRY1* [14]. Many other plant cryptochrome genes, such as *MdCRY1*, *SlCRY1*, and *BnCRY1*, promote anthocyanin accumulation as shown by increased anthocyanin levels in *Arabidopsis* seedlings transformed with these genes. Transformation with *PeCRY1* also resulted in significantly increased anthocyanin accumulation in both *cry1* mutant and WT seedlings (Fig. 6E). These results suggest that *PeCRY1* promotes anthocyanin accumulation in plants grown under blue light.

### PeCRY1 Interacts with AtCOP1 and AtSPA1

Although the signal transduction mechanism of plant cryptochromes is not fully understood, cryptochromes are generally considered to regulate plant growth and development through interactions with signaling proteins that regulate the expression of downstream genes [1], [11], [12]. In *Arabidopsis*, both CRY1 and CRY2 act largely through direct interaction with COP1 [55], [56]. Based on the highly similar structures and functions of PeCRY1 and AtCRY1, we performed bimolecular fluorescence complementation (BiFC) assays to determine whether PeCRY1 interacts with AtCOP1 and whether PeCRY1 function is conserved between poplar and *Arabidopsis*.

**Figure 7:**
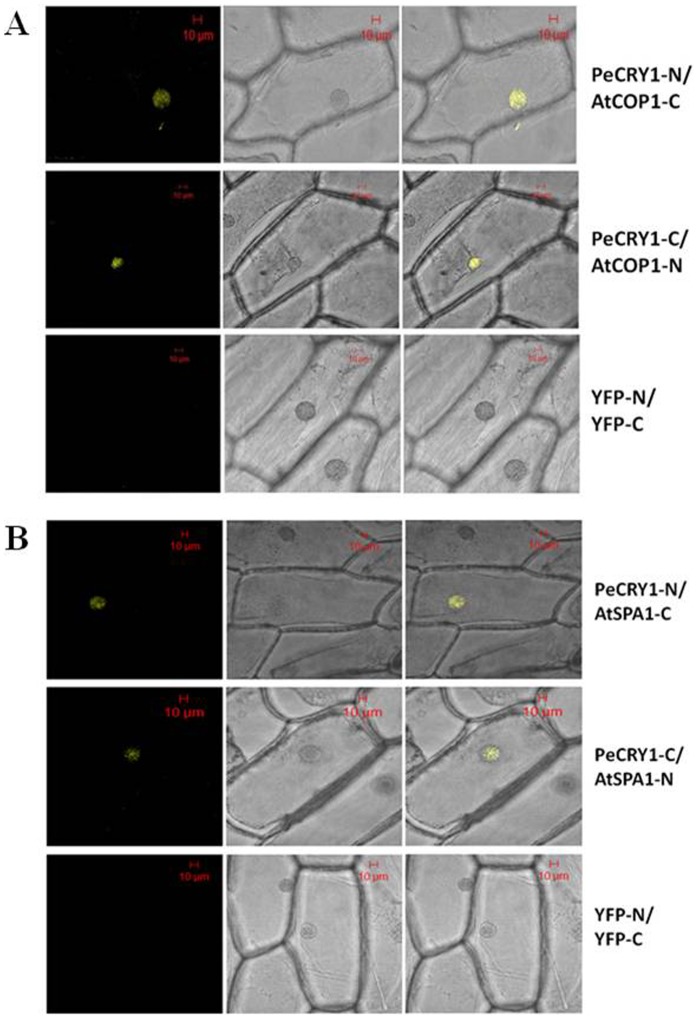
PeCRY1 interacts with AtCOP1 and AtSPA1 respectively in bimolecular fluorescence complementation (BiFc) assays.

Full-length *PeCRY1* and *AtCOP1* cDNAs were fused to the N-terminal and C-terminal halves, respectively, of yellow fluorescent protein (YFP). The fusion proteins were then introduced transiently into onion epidermal cells. Yellow fluorescence was clearly detectable when the two proteins were co-transformed, suggesting that PeCRY1 and AtCOP1 interact *in vivo* in plant cells (Fig. 7A). Besides, it has also been hypothesized that CRY1 suppress the activity of COP1 by interacting with SPA1. We found PeCRY1 also interacts with AtSPA1 just as in Arabidopsis using BiFc assays (Fig. 7B). These results provided additional evidence suggesting that PeCRY1 is a poplar counterpart of AtCRY1 and plays a role in the molecular mechanism of the light signal transduction pathway in poplar. Moreover, these results demonstrated that PeCRY1 is a nuclear-localized protein as predicted (Fig. 3C).

## Discussion

The blue light receptor cryptochromes play vital roles in plant growth and development. Studies on different species over the last 20 years indicate that cryptochromes are probably the most widely spread photoreceptors in nature and play various biological functions across the three major evolutionary lineages, bacteria, plants, and animals [35]. In this study, we cloned the cryptochrome gene *PeCRY1* from Euphrates poplar and demonstrated its function as a blue light receptor similar to that of *AtCRY1* in *Arabidopsis*.

### PeCRY1 Sequence Analysis

CRY apoprotein contains two characteristic domains: the N-terminal PHR and C-terminal CCE domains [57]. In this study, we showed that PeCRY1 also contains these conserved domains as well as the FAD-binding and C-terminal DAS domains (Fig. 1). In addition, specific amino acids and the three amino acid motifs (TGYP, WRWG, and LLDAD) located in the N-terminal PHR domain and crucial for binding with FAD or MTHF were also present in the PeCRY1 sequence (Fig. 1B). The presence of these highly conserved domains indicated that the function of PeCRY1 should be similar to other CRY proteins, especially AtCRY1.

In contrast to the conserved N-terminal PHR domain, cryptochrome C-terminal CCE domains share little sequence similarity with each other, which provides the primary means of distinguishing the cryptochromes of different plant species [35]. Based on amino acid sequence analysis using IUPred and FoldIndex software, we indentified an unstructured C-terminal region in PeCRY1 located between amino acids 480 and 650, similar to the AtCRY1 protein (Fig. 2B, C). Because unstructured regions are found frequently in signaling proteins, they have been hypothesized to be important for protein–protein interactions because they confer structural plasticity for interactions with multiple partners, more favorable energy costs for high specificity/low affinity binding of the partner proteins, and accessible posttranslational modification sites often recognized not only by signaling partners, but also by regulatory proteins [58], [59]. The CCE domain of CRY1 in *Arabidopsis* is necessary for its interaction with COP1 [55] and the interaction is light-dependent. Based on the high similarity of predicted secondary and tertiary structures between PeCRY1 and AtCRY1, we propose that PeCRY1 may be a blue light photoreceptor in Euphrates poplar that undergoes a light-induced conformational change to transmit the light signal through interaction with other proteins such as COP1 to regulate plant growth and development. The results of the BiFc assay showing that PeCRY1 can interact with AtCOP1 support this hypothesis and raise the possibility that one or more proteins like COP1 may interact with PeCRY1 in Euphrates poplar to regulate plant growth under light conditions. The identification of other proteins that can interact with PeCRY1 and how such protein–protein interactions might transmit light signals awaits further study.

### Analysis of *PeCRY1* Expression

Expression of *CRY* genes is regulated by endogenous circadian rhythms, light quality, and day length. As in *Arabidopsis*, cryptochrome mRNA levels are regulated by light in tomato [60], pea [26], [61], *Brassica* [25], and apple [29], [30]. *CRY* genes in different plant species respond differently to light induction. In garden pea, blue light inhibits *CRY* gene expression [26], but enhances *CRY1* expression in *Brassica napus* [25]. In *Arabidopsis*, the expression of cryptochrome genes was induced by blue light and exhibited an oscillation period of almost 24 h [26], [37]. In our light treatment experiments, we showed that *PeCRY1* expression is induced by blue light and that the circadian rhythm of its expression is similar to that of *AtCRY1* (Fig. 5B). In addition, analysis of *PeCRY1* expression in different Euphrates poplar tissues indicated that it was expressed constitutively (Fig. 5A) similar to *AtCRY1* expression in *Arabidopsis* [14], [15], [37] and in accordance with the various functions of cryptochrome genes in different tissues [35]. These results demonstrate the role of PeCRY1 in photoresponses and provide preliminary evidence that PeCRY1 may be a blue/UV-A light receptor in Euphrates poplar.

Studies using green fluorescent protein (GFP)/phytochrome fusion proteins have demonstrated that both phyA and phyB are expressed in *Arabidopsis* root tissues [62] as are phototropins [63] and cryptochromes [37]. In addition, both red and blue light were effective in promoting significantly increased primary root length compared to roots of dark-grown control plants [2]. In this study, we showed that PeCRY1 was expressed at a high level in Euphrates poplar root tissue and promoted primary root elongation (Fig. 6B, D). How the expression of a photoreceptor such as PeCRY1 can be induced in underground tissues without direct light is an interesting question. One possible explanation is that some light may penetrate soil to activate the three major plant photosensory receptors [64]. However, the functions of both AtCRY1 and AtCRY2 in regulating primary root elongation have been shown to be dependent on the transport of auxin hormone from the aerial part of the plant to the root tissues [2]. Studies of the last 20 years in different plant species also indicated that cryptochrome genes can regulate the synthesis, transport, or concentration of hormones such as auxin [65], [66], gibberellic acid (GA) [41], and ethylene [40]. However, if root tissue growth is regulated solely by hormones transmitted from aerial tissues, the reason for the high level of *PeCRY1* expression in root tissue remains unclear.

PHYA and PHYB proteins in root tissues have been reported to be involved in the transmission of some hormone signals from the shoot [67]. In addition, previous studies indicated that the expression of the cryptochrome gene *MdCRY2* in apple may be regulated by the hormone abscisic acid (ABA) [29]. Based on these results, we propose that in addition to their growth-regulating functions, the hormones that are regulated by cryptochrome genes and transmitted from aerial to underground tissues may promote the expression of crucial genes in roots such as *PeCRY1*. For example, we found that *PeCRY1* expression was regulated by indole acetic acid (IAA) and ABA (data not shown). Once the genes are thus induced in the roots, they can perform their functions in the same manner as in the aerial tissues, but in the absence of light. This hypothesis also leads to other questions such as whether cryptochrome expression can also be induced by hormones in the aerial tissues and whether genes expressed in underground tissues can regulate the expression of genes in the aerial tissues in the same manner. Based on the high level of *PeCRY1* expression in root tissue, we can deduce that PeCRY1 has important functions in root development such as regulating primary and lateral root elongation and the proportion of primary and lateral roots, which are important in tree growth and development.

### Functional Complementation of the *Arabidopsis cry1* Mutant

Analysis of the effects of PeCRY1 on inhibition of hypocotyl elongation, promotion of root elongation, and anthocyanin accumulation indicated that *PeCRY1* could complement the *Arabidopsis cry1* mutant phenotype (Fig. 6). These results, coupled with the expression analysis, suggest that PeCRY1 is a counterpart of AtCRY1. In addition, PeCRY1 has the two opposite functions of promoting root elongation, but inhibiting hypocotyl elongation, just as AtCRY1 does in *Arabidopsis*.

Previous studies suggested that reduced hypocotyl length resulting from blue light perception is caused primarily by a reduction in cell length and not by a reduction in cell number [68]. In addition, the effect of cryptochrome photoreceptors on root growth can be explained, to a large extent, by changes in cell length, as opposed to a change in the number of cell divisions [2]. We can conclude that at the cellular level, cryptochrome photoreceptors regulate hypocotyl and root elongation by the same mechanism. Why the same mechanism leads to opposite results remains unclear.

Auxin regulates cell elongation in numerous plant tissues, and polar auxin transport inhibitors have long been known to affect elongation growth and tropisms in roots and shoots [2], [69]. Given that cryptochrome can regulate the synthesis, transport, and concentration of hormones, we believe that cryptochrome can regulate the extent of cell elongation in different tissues by adjusting the concentration of auxin with low auxin concentration promoting growth and high auxin concentration inhibiting growth. Additional studies are needed to test this hypothesis and to fully clarify the correlation between blue light transmission and hormone signal transduction mechanisms.

## Materials and Methods

### Ethics Statement

No specific permits were required for the described field studies. The location is not privately-owned or protected in any way, and the field studies did not involve endangered or protected species.

### Plant Material And Growth Conditions

Samples of roots, stems, shoots, buds and leaves were collected from adult trees of euphrates poplar (*Populus euphratica*) in Xinjiang province, China. The poplar forest locates along the Tarim River in western China (41.0526°N; 86.2289°E).

Euphrates poplar seedlings were grown on MS medium with 0.4 mM IBA, and grown in controlled environment cabinets under 16 h light/8 h dark conditions at 23°C unless stated otherwise.

Euphrates poplar callus were grown on MS medium with 0.5 mM 2,4-D and 1.5 mM 6-BA, and grown in controlled environment cabinets in the dark at 23°C unless stated otherwise.

Columbia lines of Arabidopsis thaliana were used as the wild type (WT). Seeds of Columbia, *cry1* -mutant and transgenic lines were sown on MS medium, cold-treated for 3 days at 4°C, and then transferred to controlled environment cabinets under long days (16 h light/8 h dark) conditions at 22°C.

Experiments involving dark or different light treatments were performed in a controlled environment chamber using the blue (5 µmol m^−2^ s^−1^), red (5 µmol m^−2^ s^−1^) or white (150 µmol m^−2^ s^−1^) tubes.

### Isolation of Full-Length cDNA of *PeCRY1* by Rapid Amplification of cDNA Ends (RACE)

An EST sequence (AJ768957) with 581 bp in length encoding *PeCRY1* was isolated though RT-PCR using the degenerate primer pair Y1EST-F (5′-TCTTGGTTGGCAATACATAACC-3′) and Y1EST-R (5′- TTGGTTGTGGACTGACATT-3′). To obtain the full-length gene, 5′- and 3′-RACE was used. Total RNA was isolated from euphrates poplar leaves using the Trizol (Invitrogen, Carlsbad, CA, USA) method according to the manufacturer's instructions. The 5′-RACE primers Y1-5-R1 (5′-GCACGTATTCTCCATTTGGGTC-3′) and Y1-5-R2 (5′-GGTTATGTATTGCCAACCAAG -3′), and the 3′-RACE primers Y1-3-F1 (5′-GACGAAGAAGCTTCTTCAG-3′) and Y1-3-F2 (5′-GATGTAAATGTCAGTCCAC-3′) were designed on the basis of the *PeCRY1* EST sequence. The PCR products of expected sizes were purified, cloned into the pMD18-T vector (Takara, Otsu, Japan), and sequenced. Then, the putative 3′-and 5′-RACE cDNAs and the EST sequence were over-lapped with DNAMAN to form a cDNA contig, which was used to determine the putative initiating translation codon (ATG) and open reading frame (ORF). To obtain a full-length cDNA of *PeCRY1*, a pair of full-length primers PeCRY1-F (5′-ATGTCAGGAGGTGGGTGTAG-3′) and PeCRY1-R (5′-TTACCCGGTTTGGGGTAGCC-3′) were designed according to the contig. The full length of *PeCRY1* was then obtained by RT-PCR using the full-length primers.

### Expression Analysis

RNA was extracted using TRizol Reagent (Invitrogen, Carlsbad, CA, USA), then reverse transcribed using the PrimeScript First Strand cDNA Synthesis Kit (Takara, Otsu, Japan), following the manufacturer's instructions. 10 µl of cDNA was diluted to a final volume of 100 µl with water.

Semiquantitative RT-PCR was carried out in 25-µl reactions with 5 ng diluted cDNA template. The PCR profile was 94°C for 3 min, 30 cycles of 94°C for 30 s, 56°C for 30 s, and 72°C for 30 s, with a 5 min extension at 72°C. The primers used were bdlY1-F (5′-CTGCTACAAATCGCCGCTAC-3′) and bdlY1-R (5′-CATCACCAACAAACTGCTCTG-3′). *PeACT* cDNA amplification was used as the external control, and primers used were PeACT-F (5′-GTCCTCTTCCAGCCATCTC-3′) and PeACT-R (5′-TTCGGTCAGCAATACCAGG-3′). PCR products were electrophoresed on a 1.5% agarose gel and viewed under UV light after standard staining with ethidium bromide.

For real-time quantitative RT-PCR analysis, the specific primer used were DLY1-F (5′-GTCCCTTCACAACCTTTGCT-3′) and DLY1-R (5′-GCATCACCCGAAATAATCCT -3′). Poplar actin (*PeACT*) gene was used as loading controls. Fluorescence-quantitative PCR reactions were repeated three times.

### Generation of *PeCRY1*-Transgenic Arabidopsis Plants

To generate Arabidopsis *PeCRY1* overexpressing lines, the full length of *PeCRY1* was amplified by PCR using the primers PeCRY1-F and PeCRY1-R. The amplified cDNA was cloned into the expression vector pRI (pRI 101-AN) under the control of the cauliflower mosaic virus (CaMV)-35S promoter. The plasmids were then transformed into WT and *cry1* mutant *Arabidopsis* separately, mediated by *Agrobacterium* GV3101 using the floral dip method (Clough and Bent 1998).

### Hypocotyl And Root Measurements

For hypocotyl and root growth experiments, hypocotyl and root lengths of at least 30 Arabidopsis seedlings grown in appointed conditions were measured. Experiments were performed in at least three independent biological replicates.

### Measurement of the Total Anthocyanin Concentration

Total anthocyanin was extracted according to methanol–HCl method, in which the samples were extracted independently overnight in 5 ml methanol and 1% (v/v) HCl with extraction at room temperature. The absorbance of each extract was measured at 530, 620 and 650 nm with a spectrophotometer (UV-1600, Shimadzu, Kyoto, Japan). The relative anthocyanin content was determined by the formula OD =  (A_530_ - A_620_) - 0.1(A_650_ - A_620_). One unit of anthocyanin content was expressed as a change of 0.1 OD (unit ×10^3^/g FW).

### Bimolecular Fluorescence Complementation (BiFC)

The full-length *PeCRY1* and *AtCOP1* cDNAs were cloned into pYFP-N (1–155) and pYFP-C (156–239) vectors and sequenced. Onion epidermal cells were transiently transformed using the *Agrobacteriumin* fection method with different combinations of these constructs. YFP-dependent fluorescence was detected 24 h after transfection using a confocal laser scanning microscope (Carl Zeiss; LSM 510 Meta).
